# Long-term clinical outcomes of hematopoietic cell transplantation for intermediate-to-poor-risk acute myeloid leukemia during first remission according to available donor types

**DOI:** 10.18632/oncotarget.15295

**Published:** 2017-02-11

**Authors:** Jae-Ho Yoon, Hee-Je Kim, Sung-Soo Park, Young-Woo Jeon, Sung-Eun Lee, Byung-Sik Cho, Ki-Seong Eom, Yoo-Jin Kim, Seok Lee, Chang-Ki Min, Seok-Goo Cho, Dong-Wook Kim, Jong-Wook Lee, Woo-Sung Min

**Affiliations:** ^1^ Department of Hematology, Catholic Blood and Marrow Transplantation Center, Leukemia Research Institute, Seoul St. Mary’s Hospital, College of Medicine, The Catholic University of Korea, Seoul, Korea

**Keywords:** acute myeloid leukemia, allogeneic hematopoietic cell transplantation, autologous hematopoietic cell transplantation, familial mismatched hematopoietic cell transplantation

## Abstract

Standard therapy for acute myeloid leukemia (AML) consists of hematopoietic cell transplantation (HCT) including autologous-HCT (AUTO) and allogeneic-HCT from a matched-sibling donor (MSD) or well-matched unrelated donor (WM-URD). When a conventional donor is not available, HCT from a partially-matched (PM)-URD or familial-mismatched donor (FMMD) is typically considered. We analyzed 561 patients with intermediate to poor-risk molecular cytogenetics who underwent transplant from 2002 to 2013 in their first remission. Engraftment was successful in all donor types except five patients who died in aplasia. Disease-free survival (DFS) at 5 years was 61.4% for MSD, 62.1% for WM-URD, 65.3% for FMMD, 44.7% for AUTO and 36.8% for PM-URD. AUTO showed the highest relapse rate (51.0%) compared to MSD (23.5%) and FMMD (18.5%), but showed the lowest 5-year non-relapse mortality (NRM) rate (3.8%). PM-URD showed the highest NRM (29.3%) with more instances of acute graft-vs.-host disease (GVHD) with grade≥III (29.3%), compared to MSD (15.6%) and FMMD (15.7%). In a poor-risk subgroup, the 5-year DFS for FMMD and MSD was 59.8% and 46.7%, respectively, while for AUTO and PM-URD it was 12.6% and 0.0%, respectively, which was caused by a high relapse rate (87.1% in AUTO, 83.3% in PM-URD). In the intermediate-risk subgroup, the 5-year DFS of AUTO (53.9% was not different from the conventional donors in multivariate analysis, presenting a low NRM rate (5.1%). FMMD should be considered prior to PM-URD in intermediate-to-poor-risk AML and GVHD prophylaxis should be intensified when PM-URD is needed. AUTO might be considered for selected patients in the intermediate-risk group.

## INTRODUCTION

Standard therapy for acute myeloid leukemia (AML) consists of several hematopoietic cell transplantation (HCT) strategies including autologous (AUTO)-HCT, allogeneic-HCT from a matched-sibling donor (MSD), or well-matched unrelated donor (WM-URD) transplantation. Although many reports show positive results in the favorable-risk group [1–5] and in selected patients with non-favorable karyotypes [6–10], the role of AUTO-HCT still remains unclear, and the procedure fell out of fashion in clinical trials. In contrast, allogeneic-HCT is regarded as a curative option for adult AML patients in their first complete remission (CR1), especially in the intermediate- and poor-risk groups, which showed lower relapse rates showing graft-versus-leukemia (GVL) effect compared to AUTO-HCT or chemotherapy alone [11–13]. However, only 30% of patients have human leukocyte antigen (HLA)-identical sibling donors available [14, 15], and therapy-related mortality caused by complications from infections or acute/chronic graft-versus-host disease (GVHD) is still an important challenge in allogeneic-HCT [16]. When searching for HCT donors, if a MSD is not available, a WM-URD is another standard choice since the survival outcomes are comparable due to the development of immunosuppressive agents [17, 18]. When neither standard HCT donors are available, alternative donor types, such as a partially-matched unrelated donor (PM-URD) or a familial mismatched/haploidentical transplantation (FMMT), are now being considered [19].

Previous data showed that HLA-mismatch was associated with more transplantation-related complications and poorer survival outcome caused by higher rates of graft failure and higher-grade GVHD [20–22]. However, many recent studies have shown that the outcomes of FMMTs have been significantly improved by optimization of pre-conditioning regimens and the development of modalities for immunosuppression to overcome major HLA barriers [23–27], while PM-URD is still controversial as a viable alternative [18, 28, 29]. In addition, HLA-mismatch can potentially induce natural killer (NK)-cell alloreactivity, which may reduce relapse rates of AML [30, 31]. For FMMT specifically, we can expect prompt donor availability, preferred graft control, and a timely application of repeated donations, which may be large advantages to poor-risk AML patients who are in urgent need of allogeneic-HCT [14, 15]. Despite the positive expectations for FMMT, few prospective studies have analyzed survival outcomes compared to standard donor types, and true randomized studies cannot be conducted for ethical reasons.

We have previously analyzed the clinical outcomes of FMMT compared to WM-URD and PM-URD in a pilot study with a small number of patients and a short follow-up duration [19]. In the current study, we analyzed the long-term HCT outcomes of several donor types, including AUTO-HCT, MSD, WM-URD, PM-URD and FMMT, in intermediate- to poor-risk AML patients in CR1.

## RESULTS

### Baseline characteristics

**Figure 1:**
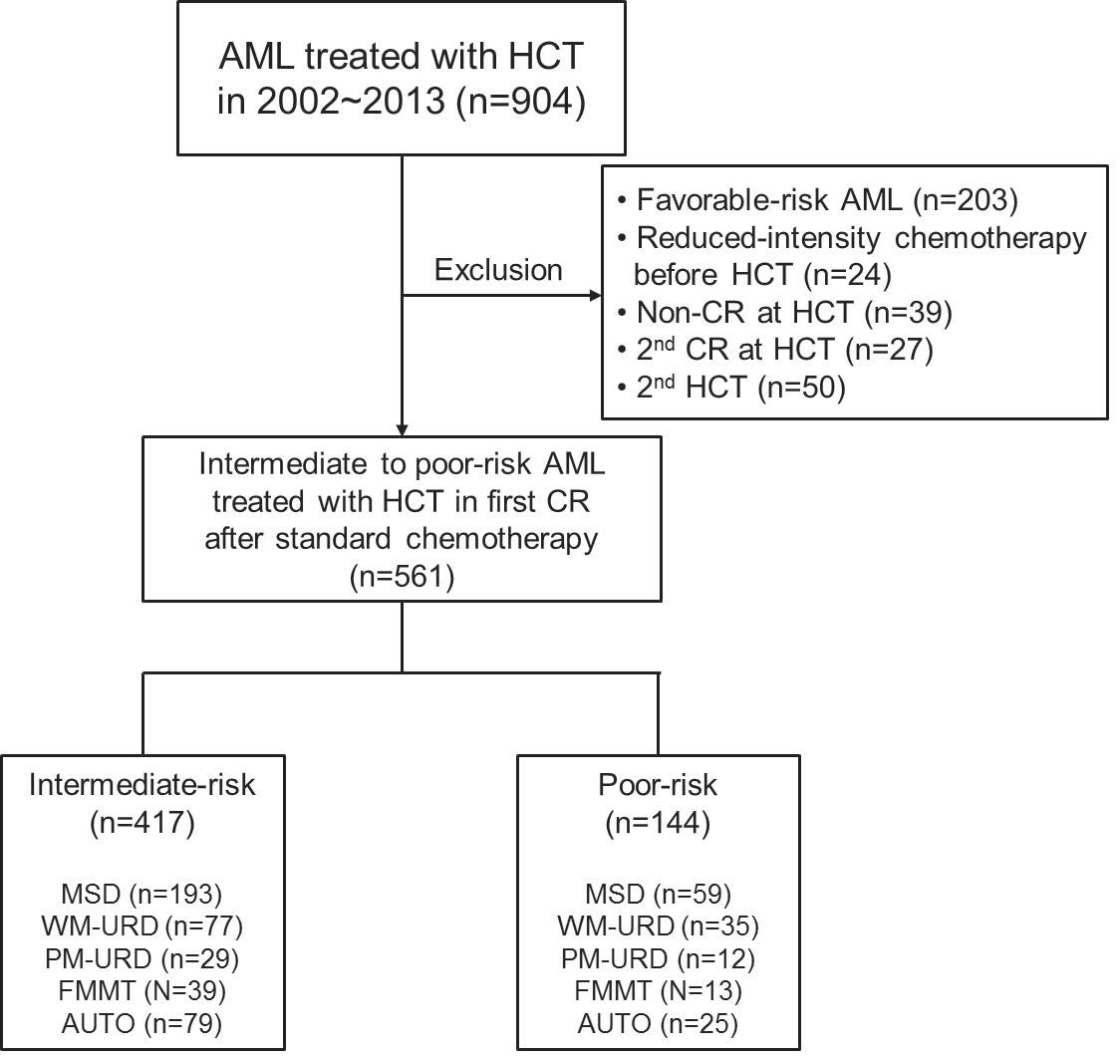
Consort diagram of analyzed patients in the current study Abbreviation: AML, Acute myeloid leukemia; HCT, hematopoietic cell transplantation; CR, complete remission; MSD, matched sibling donor; WM-URD, well matched unrelated donor; PM-URD, partially matched unrelated donor; FMMT, familial mismatched transplantation; AUTO, autologous HCT

A consort diagram of the finally-selected 561 patients with intermediate- to poor-risk molecular cytogenetics is presented in Figure 1. All 561 patients underwent HCT in the first remission after intensive chemotherapy and the baseline characteristics are described in Table 1. Among them, there were 417 patients in the intermediate-risk group and 144 patients in the poor-risk group (Figure 1). The median age was 40 years old (range, 16-68 years old) and the median time to HCT was 5.5 months (range, 3.0-11.5 months). In 144 (25.7%) patients, the time to HCT exceeded 7 months. Among them, 68 patients had a slight delay (within 7.5 months) mainly due to the lack of transplantation facilities, 19 patients underwent numerous chemotherapy cycles (> 3 cycles with 2 month-interval) due to a delayed first CR, 10 patients had a delayed donor search, and 47 patients suffered from either delayed neutrophil recovery or infectious complications after prior chemotherapy. Among patients with core-binding factor-positive AML, *c-kit* mutations were identified in 30 patients who were then included in the intermediate-risk group. Among the 356 cytogenetically normal (CN)-AML patients, 64 (18.0%) with an isolated *NPM1* mutation were classified into the favorable-risk group and excluded initially, while 137 (38.5%) patients without available molecular data and 120 (33.7%) *NPM1*-negative/*FLT3*-ITD-negative patients were classified into the intermediate-risk group. Thirty-five CN-AML patients with *FLT3*-ITD mutations (10.0%) were classified into the poor-risk group. For pre-transplant chemotherapy, 60.2% of patients were treated with an N^4^-behenoyl-1-β-D-arabinofuranosyl cytosine- (BHAC) based regimen and 39.8% of patients were treated with a cytosine arabinoside- (ARA-C) based regimen. AUTO was performed in 104 (18.5%) patients, MSD-HCT in 252 (44.9%) patients, URD-HCT in 153 (27.3%) patients, and FMMT in 52 (9.3%) patients. Among the patients treated with URD-HCT, 112 patients received stem cells from WM-URD and 41 from PM-URD. Almost half of the patients (50.3%) were treated with cyclophosphamide plus 1320 cGy of total body irradiation (TBI), while a reduced-intensity conditioning (RIC) regimen was used in 8.2% of patients, and 9.3% of patients were treated with an FMMT regimen.

**Table 1:** Baseline characteristics of the entire patients

	N (Total 561 patients)
Age, median (range)	40.0 years old (16-68)
Gender, male (%)	295 (52.5%)
Leukocyte (x10^9^/L), median (range)	11.8 (0.5-835.0)
Platelet (x10^9^/L), median (range)	54.5 (5.0-882.0)
PB blast (%), median (range)	42.0 (0-98)
Diagnosis to HCT, median (range)	5.5 months (3.0-11.5)
< 7 months	417 (74.3%)
≥ 7 months	144 (25.7%)
WHO classification	
Recurrent genetic abnormality	
t(8;21)(q22;q22) / inv(16)(p13.1q22)	22 (3.9%) / 8 (1.4%)
t(9;11)(p22;q23)	12 (2.1%)
t(6;9)(p23;q34) / inv(3)(q21q26..2)	9 (1.6%) / 4 (0.7%)
AML MRC	58 (10.4%)
Therapy-related AML	5 (0.9%)
AML M0	22 (3.9%)
AML M1	127 (22.6%)
AML M2	155 (27.6%)
AML M4	60 (10.7%)
AML M5	49 (8.%)
AML M6	22 (3.9%)
AML M7	7 (1.2%)
Acute panmyelosis with myelofibrosis	1 (0.2%)
Molecular cytogenetics (NCCN)	
Intermediate-risk (n=417)	
CN-AML (molecular data unavailable)	137 (24.4%)
*FLT3-ITD*(-) CN-AML	120 (21.4%)
*c-kit* positive CBF-AML	30 (5.3%)
Others	130 (23.2%)
Poor-risk (n=144)	
*FLT3-ITD*(+) CN-AML	35 (6.2%)
Others	109 (19.4%)
Induction	
Idarubicin/BHAC 3+7	338 (60.2%)
Idarubicin/ARA-C 3+7	223 (39.8%)
Post-remission therapy	
Autologous HCT	104 (18.5%)
Allogeneic HCT	
Matched sibling donor	252 (44.9%)
Unrelated donor	153 (27.3%)
Familial mismatched donor	52 (9.3%)
HCT conditioning regimen	
MAC (n=463)	
Cyclophosphamide/TBI 1320 cGy	282 (50.3%)
Busulfan/TBI 1320 cGy	16 (2.9%)
Busulfan/Cyclophosphamide	44 (7.8%)
Cytarabine/Melphalan/TBI 1200 cGy	121 (21.6%)
RIC (n=98)	
Busulfan/Fludarabine/TBI 400 cGy	46 (8.2%)
Busulfan/Fludarabine/TBI 800 cGy	52 (9.3%)
HCT source	
BM	225 (40.1%)
PB	324 (57.8%)
BM+PB	12 (2.1%)

Abbreviation: PB, peripheral blood; BM bone marrow; AML, acute myeloid leukemia; MRC, myelodysplasia-related change; CN, cytogenetically normal; CBF, core-binding factor positive; BHAC, N4-behenoyl-1-β-D-arabinofuranosyl cytosine; ARA-C, cytosine arabinoside; HCT, hematopoietic cell transplantation; MAC, myeloablative conditioning; RIC, Reduced intensity conditioning; TBI, total body irradiation;

Pre- and post-HCT parameters are presented in Table 2 according to HCT donor type. Between the subgroups, there were more female patients in the MSD subgroup, and the MSD and WM-URD subgroups had more patients who underwent HCT within 7 months. Blood cell and blast counts, as well as the molecular cytogenetic risk groups were not significantly different between the 5 subgroups. FMMT and AUTO were conducted using the same HCT conditioning regimen, while MSD- and URD-HCT were conducted using variable regimens including myeloablative conditioning (MAC) and RIC. The MAC regimen was used in 88.9% of cases for MSD, 85.7% for WM-URD, and 95.1% for PM-URD. Among the MAC regimens, TBI-based conditioning regimens were used more often with MSD-HCT compared to URD-HCT (92.9% *vs*. 79.3%, *p* = 0.001). BM was more often used for the stem cell source in MSD-HCT compared to URD-HCT (71.1% *vs*. 36.6%, *p* < 0.001), while all of the FMMT and AUTO procedures were conducted with PB as the stem cell source. The CD34+ stem-cell dose was significantly higher in FMMT, while the CD3+ dose was significantly lower in MSD-HCT compared to the other donor types.

**Table 2:** Baseline characteristics of AML patients who achieved CR after standard chemotherapy

	MSD (n=252)	WM-URD (n=112)	PM-URD (n=41)	FMMT (n=52)	AUTO (n=104)	*p*
Age, median (range)	41.0 (16-68)	37.0 (16-68)	42.0 (16-60)	41.0 (16-64)	39.0 (16-67)	0.158
Gender, male (%)	112 (44.4%)	70 (62.5%)	24 (58.5%)	30 (57.7%)	59 (56.7%)	0.011*
Leukocyte (x10^9^/L)	12.4 (0.5-376.2)	12.7 (1.2-835.0)	11.6 (0.9-123.9)	11.1 (0.8-395.9)	11.5 (0.9-308.4)	0.759
Hemoglobin (g/dL)	8.6 (3.5-16.8)	9.0 (2.0-14.8)	8.9 (3.5-14.7)	9.3 (5.3-16.1)	8.6 (4.7-13.4)	0.386
Platelet (x10^9^/L)	56.0 (5.0-408.0)	56.0 (5.0-272.0)	53.0 (9.0-682.0)	55.0 (5.0-629.0)	42.0 (5.0-491.0)	0.129
PB blast (%)	45.5 (0-98)	32.0 (0-98)	42.0 (0-98)	47.5 (0-94)	40.5 (0-98)	0.369
BM blast (%)	84.0 (6-99)	81.5 (20-99)	85.0 (20-98)	87.5 (20-98)	84.5 (16-99)	0.749
Diagnosis to HCT						
< 7 months	224 (88.9%)	79 (70.5%)	24 (58.5%)	33 (63.5%)	57 (54.8%)	<0.001*
≥ 7 months	28 (11.1%)	33 (29.5%)	17 (41.5%)	19 (36.5%)	47 (45.2%)	
Molecular Cytogenetics						
Intermediate (n=417)	193 (76.6%)	77 (68.8%)	29 (70.7%)	39 (75.0%)	79 (76.0%)	0.569
CN-AML	117 (46.4%)	38 (33.9%)	17 (41.5%)	22 (42.3%)	63 (60.6%)	
*c-kit*(+) CBF-AML	16 (6.4%)	5 (4.5%)	2 (4.8%)	5 (9.6%)	2 (1.9%)	
Others	60 (23.8%)	34 (30.3%)	10 (24.4%)	12 (23.1%)	14 (13.4%)	
Poor-risk (n=144)	59 (23.4%)	35 (31.2%)	12 (29.3%)	13 (25.0%)	25 (24.0%)	0.569
*FLT3*(+) CN-AML	19 (7.5%)	10 (8.9%)	0 (0.0%)	3 (5.8%)	3 (2.9%)	
Others	40 (15.9%)	25 (22.3%)	12 (29.3%)	10 (19.2%)	22 (21.1%)	
Induction						
IDA/BHAC 3+7	153 (60.7%)	65 (58.0%)	28 (68.3%)	18 (34.6%)	74 (71.2%)	<0.001*
IDA/ARA-C 3+7	99 (39.3%)	47 (42.0%)	13 (31.7%)	34 (65.4%)	30 (28.8%)	
Conditioning regimen						
TBI ≥ 800cGy (n=463)						
CY/TBI 1320 cGy	192 (76.2%)	65 (58.1%)	25 (60.9%)	0	0	0.001*^†^
BU/TBI 1320 cGy	13 (5.2%)	2 (1.8%)	1 (2.4%)	0	0	0.273^†^
TAM 1200cGy	3 (1.2%)	8 (7.1%)	6 (14.6%)	0	104 (100%)	0.001*^†^
BU/FLU/TBI 800cGy	0	0	0	52 (100%)	0	NA
TBI < 800 cGy (n=98)						
BU/CY	16 (6.3%)	21 (18.8%)	7 (17.1%)	0	0	0.001*^†^
BU/FLU/TBI 400cGy	28 (11.1%)	16 (14.3%)	2 (4.9%)	0	0	0.262^†^
HCT source						
BM	179 (71.1%)	39 (34.8%)	17 (41.5%)	0 (0.0%)	0 (0.0%)	<0.001*^†^
PB	69 (27.4%)	73 (65.2%)	24 (58.5%)	52 (100%)	96 (92.3%)	
BM+PB	4 (1.5%)	0 (0.0%)	0 (0.0%)	0 (0.0%)	8 (7.7%)	
CD34+ stem cells	3.26 (0.47-49.00)	4.7 (0.17-27.39)	4.7 (0.68-34.44)	6.34 (0.63-17.1)	3.76 (0.10-24.87)	<0.001*
CD3+ stem cells	49.43 (0.6-986.1)	244.7 (1.4-854.2)	254.2 (3.4-666.9)	408.3 (3.9-937.2)	310 (75.0-774.2)	<0.001*

Subgroup was made according to the HCT donor source.

†*p*-value analyzed between the first three groups except for FMMT and AUTO.

*p< 0.005

Abbreviation: PB, peripheral blood; BM bone marrow; HCT, hematopoietic cell transplantation; CN, cytogenetically normal; CBF, core-binding factor positive; BHAC, N^4^-behenoyl-1-β-D-arabinofuranosyl cytosine; ARA-C, cytosine arabinoside; TBI, total body irradiation; CY, cyclophosphamide; BU, busulfan; TAM, TBI plus ARA-C plus melphalan; FLU, fludarabine;

### Clinical outcomes

**Figure 2:**
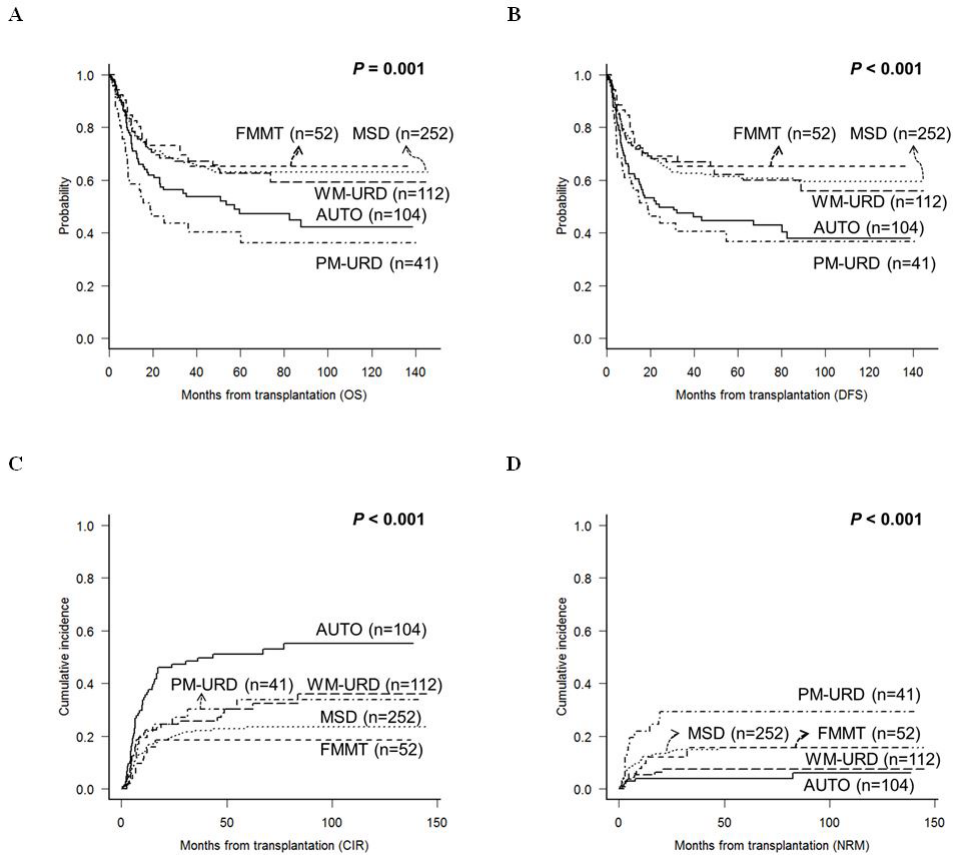
Treatment outcomes of total 561 patients according to the transplantation donor sources **A**. OS. **B**. DFS. **C**. CIR. **D**. NRM.

Engraftment was successful in all patients, except for 5 patients who died either from aplasia due to infectious complications or from sudden cardiac death. The median time to neutrophil-count recovery was 1 day faster in FMMT (11 days, range: 10-17) compared to AUTO (12 days, range: 7-78), MSD (12 days, 2-30), WM-URD (12 days, range: 6-29), and PM-URD (12 days, range: 10-21). The median time to platelet-count recovery was longer in MSD (15 days, range: 0-44) and PM-URD (13 days, range: 5-45) compared to FMMT (12 days, range: 0-21), AUTO (11 days, range: 0-123), and WM-URD (12 days, range: 0-50). However, after adjusting for pre-conditioning intensity and stem cell sources, recovery times were not significantly different between the donor types. Our data shows that ABO mismatch, sex mismatch, certain HLA locus mismatches between the donor and recipient, and stem cell source were not influential for either survival outcome or the incidence rate of acute and chronic GVHD. At the time of data analysis, 218 of the 561 patients had died, with 148 from leukemia relapse and 70 from other causes. The most common cause of death was pneumonia (*n* = 22) from variable causes - invasive aspergillosis (*n* = 6), *Pneumocystis jirovecii* pneumonia (*n* = 4), CMV pneumonia (*n* = 2), idiopathic (*n* = 10) - followed by acute gut GVHD (*n* = 11), veno-occlusive disease (*n* = 7), sepsis (*n* = 6), acute hepatic GVHD (*n* = 5), chronic lung GVHD (*n* = 5), thrombotic microangiopathy (*n* = 4), and hemorrhagic cystitis (*n* = 4), acute renal failure (*n* = 2), hepatitis B reactivation (*n* = 2), sudden cardiac death (*n* = 1), and lymphoma (*n* = 1). After a median follow-up duration of 58.7 months (range: 9.6-150.6), the 5-year overall survival (OS) of MSD, WM-URD, and FMMT was 63.1%, 63.9%, and 65.1%, respectively, while the rates for AUTO and PM-URD, 47.2% and 40.3%, respectively, were significantly inferior (Figure 2A). The disease-free survival (DFS) rate at 5 years was 61.4% for MSD, 62.1% for WM-URD, 65.3% for FMMT, 44.7% for AUTO, and 36.8% for PM-URD (Figure 2B). The 5-year cumulative incidence of relapse (CIR) rate was highest in AUTO (51.0%), followed by PM-URD (33.9%), WM-URD (30.3%), MSD (23.5%), and FMMT (18.5%) (Figure 2C). In contrast, the lowest 5-year non-relapse mortality (NRM) rate was identified in AUTO (3.8%), followed by WM-URD (7.4%), FMMT (15.7%), and MSD (15.6%). PM-URD showed the highest 5-year NRM rate at 29.3% (Figure 2D). Overall, MSD, WM-URD, and FMMT showed similar 5-year OS (63.1%) and DFS (62.0%) rates, which were significantly superior to AUTO (*p* = 0.006 and *p* < 0.001, respectively) and PM-URD (*p* < 0.001 and *p* < 0.001, respectively). PM-URD had a higher NRM rate compared to AUTO (*p* < 0.001), WM-URD (*p* < 0.001), FMMT (*p* = 0.079), and MSD (*p* = 0.043). Except for AUTO-HCT, the other donor types did not show significantly-different CIR rates (e.g., FMMT *vs*. PM-URD, *p* = 0.219).

### GVHD and cytomegalovirus (CMV) reactivation

**Figure 3:**
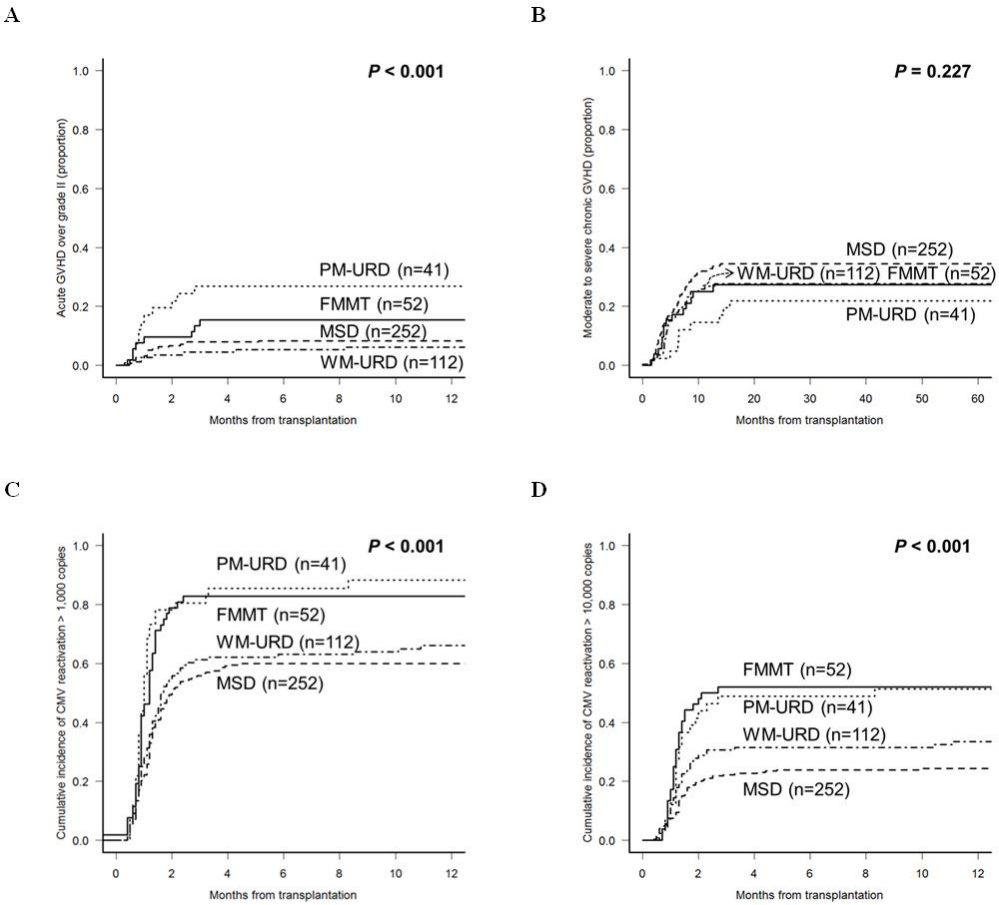
Cumulative incidence of post-HCT complications according to the donor sources **A**. Acute GVHD over grade II. **B**. Moderate to severe chronic GVHD. **C**. CMV reactivation over 1,000 copies/mL. **D**. CMV reactivation over 10,000 copies/mL.

We analyzed the incidence of GVHD and CMV reactivation according to the donor types, except for AUTO-HCT. The incidence of acute GVHD ≥ grade II was higher in URD-HCT (48.8% in PM-URD and 47.3% in WM-URD) compared to FMMT (36.5%, *p* = 0.023) and MSD-HCT (34.6%, *p* = 0.021). However, the incidence of acute GVHD ≥ grade III was higher in PM-URD (29.3%) and FMMT (15.4%) compared to MSD-HCT (8.7%) and WM-URD (6.3%). As shown in Figure 3A, PM-URD had a significantly higher proportion of acute GVHD ≥ grade III compared to WM-URD and MSD (*p* < 0.001). The incidence of chronic GVHD was similar in all donor types (55.2% in MSD, 45.8% in WM-URD, 41.5% in PM-URD, and 56.3% in FMMT). Moderate to severe chronic GVHD also occurred at a similar rate in MSD (34.4%), WM-URD (27.8%), PM-URD (22.0%), and FMMT (27.3%) without a significant difference (Figure 3B, *p* = 0.227). We analyzed the incidence of CMV reactivation (defined as more than 1,000 copies/mL) and identified that PM-URD and FMMT showed a higher incidence of CMV reactivation compared to MSD and WM-URD (88.3% and 82.7% *vs*. 62.0% and 66.0%, respectively, *p* < 0.001) (Figure 3C). When CMV reactivation was defined as more than 10,000 copies/mL (Figure 3D), PM-URD and FMMT also showed a higher incidence of CMV reactivation compared to MSD and WM-URD (51.4% and 54.4% *vs*. 25.3% and 33.5%, respectively, *p* < 0.001).

### Subgroup analysis

**Figure 4:**
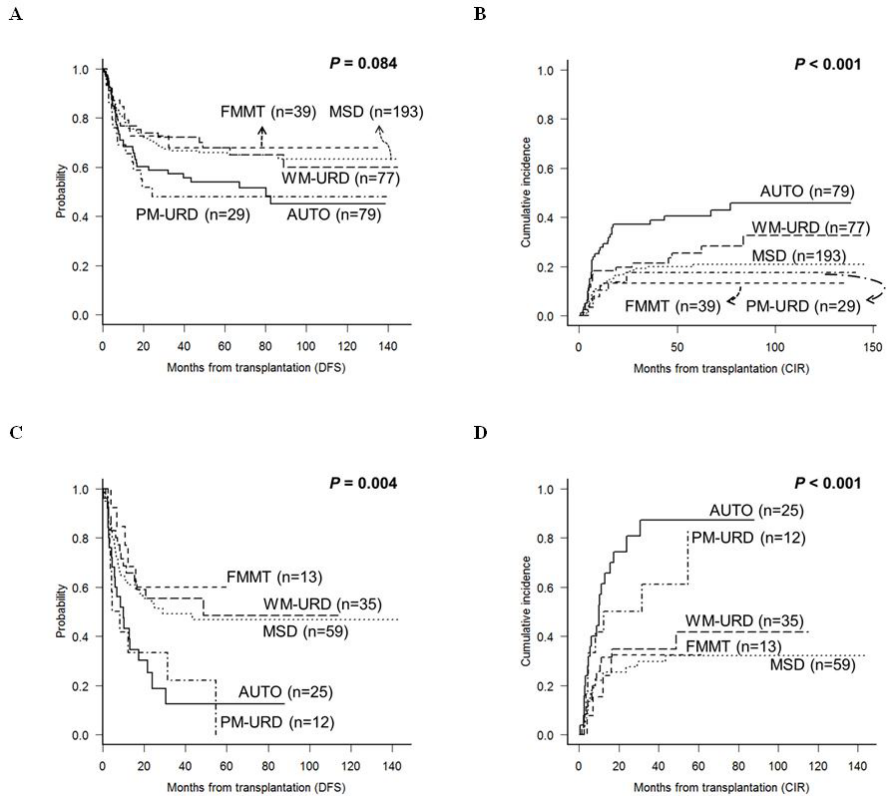
DFS and CIR rates according to the transplantation donor sources in the subgroup analysis **A**. & **B**. Intermediate-risk subgroup. **C**. & **D**. Poor-risk subgroup.

In the intermediate-risk subgroup, the 5-year DFS for PM-URD and AUTO-HCT was 48.0% and 53.9%, respectively, which was inferior to MSD (65.9%, *p* = 0.055 for PM-URD and *p* = 0.031 for AUTO-HCT), FMMT (68.5%, *p* = 0.097 for PM-URD and *p* = 0.176 for AUTO-HCT), and WM-URD (80.9%, *p* = 0.080 for PM-URD and *p* = 0.081 for AUTO-HCT) (Figure 4A). For CIR (Figure 4B) and NRM rates, only AUTO-HCT showed a significantly-higher 5-year CIR rate (40.6%) compared to other donor types (range, 13.4%-25.6%, *p* = 0.058 to 0.007), and only PM-URD showed a higher 5-year NRM rate (34.5%) compared to other donor types (range, 5.1%-18.0%, *p* = 0.093 to < 0.001). PM-URD showed high incidences of both acute GVHD ≥ grade III (31.0%) and CMV reactivation of over 10,000 copies/mL (45.1%) in the intermediate-risk subgroup. In the poor-risk subgroup, the 5-year DFS for PM-URD and AUTO-HCT was 12.6% and 0.0%, respectively, which was inferior to MSD (46.7%, *p* = 0.028 for PM-URD and *p* = 0.007 for AUTO-HCT), FMMT (59.8%, *p* = 0.031 for PM-URD and *p* = 0.015 for AUTO-HCT), and WM-URD (48.4%, *p* = 0.018 for PM-URD and *p* = 0.006 for AUTO-HCT) (Figure 4C). We also identified that AUTO-HCT had a higher 5-year CIR rate (87.1%) compared to MSD (32.1%, *p* < 0.001), WM-URD (41.8%, *p* < 0.001), and FMMT (32.5%, *p* = 0.005), but the rate was not significantly higher than PM-URD (83.3%, *p* = 0.235) (Figure 4D). The CIR rate for PM-URD was significantly higher only compared to MSD (*p* = 0.016).

### Multivariate analysis

Table 3 shows an analysis of the independent factors affecting DFS and CIR rates after HCT in the first remission. Univariate analysis showed that age, gender, and differences in pre-HCT chemotherapy did not affect DFS or CIR in this cohort. For both DFS and CIR, the proven adverse factors were the poor-risk karyotype, more than 2 induction chemotherapies to achieve first remission, time to HCT, conditioning intensity, donor types (PM-URD or AUTO-HCT), acute GVHD (only for CIR), and the absence of chronic GVHD, which were all adjusted for in multivariate analysis to a significance of *p* < 0.100. The multivariate analysis revealed that poor DFS was associated with the poor-risk karyotype (HR = 1.807 (95% CI, 1.4-2.4), *p* < 0.001), more than 2 induction chemotherapies to achieve first remission (HR = 1.567 (95% CI, 1.1-2.3), *p* = 0.017), PM-URD (HR = 1.638 (95% CI, 1.0-2.6), *p* = 0.033), and the absence of chronic GVHD (HR = 1.649 (95% CI, 1.2-2.3), *p* = 0.005). Higher CIR rates were associated with the poor-risk karyotype (HR = 2.105 (95% CI, 1.5-2.9), *p* < 0.001), more than 2 induction chemotherapies to achieve first remission (HR = 1.649 (95% CI, 1.1-2.6), *p* = 0.027), AUTO-HCT (HR = 1.962 (95% CI, 1.3-3.0), *p* = 0.002), and the absence of chronic GVHD (HR = 2.704 (95% CI, 1.6-4.5), *p* < 0.001). The risk factors for a higher rate of NRM were an age of more than 40 years old (HR = 1.729 (95% CI, 1.1-2.8), *p* = 0.023), acute GVHD ≥ Grade III (HR = 2.483 (95% CI, 1.5-4.0), *p* < 0.001), and PM-URD (HR = 3.781 (95% CI, 1.3-11.2), *p* = 0.016, compared to AUTO-HCT).

**Table 3:** Multivariate analysis of affecting factors for DFS and CIR

Variables	Disease free survival (DFS)				Cumulative incidence of relapse (CIR)			
	Univariate		Multivariate		Univariate		Multivariate	
	5-year DFS	*p*	HR (95% CI)	*p*	5-year CIR	*p*	HR (95% CI)	*p*
Age at diagnosis								
≤ 40 years old (n=283)	57.9%	0.458			32.8%	0.217		
> 40 years old (n=278)	55.4%				28.6%			
Gender								
Male (n=295)	53.9%	0.345			31.5%	0.913		
Female (n=266)	59.8%				29.7%			
Karyotype								
Intermediate-risk (n=417)	62.7%	<0.001*	1	-	24.8%	<0.001*	1	-
Poor-risk (n=144)	38.7%		1.807 (1.4-2.4)	<0.001*	48.1%		2.105 (1.5-2.9)	<0.001*
Pre-HCT chemotherapy								
Idarubicin+BHAC (n=338)	59.4%	0.282			28.2%	0.169		
Idarubicin+cytarabine (n=223)	51.0%				34.8%			
Number of inductions to CR								
1 cycle (n=491)	59.3%	0.003*	1	-	28.9%	0.071	1	-
2 or more cycles (n=70)	39.4%		1.567 (1.1-2.3)	0.017*	42.8%		1.649 (1.1-2.6)	0.027*
Time from induction to HCT								
< 7 months (n=417)	60.0%	<0.001*			27.7%	0.002*		
≥ 7 months (n=144)	46.8%				39.1%			
Conditioning regiment intensity								
MAC (n=463)	55.1%	0.081			32.8%	0.006*		
RIC (n=98)	64.9%				20.3%			
Donor source								
MSD (n=252)	61.4%	0.001*	1	-	23.5%	<0.001*	1	-
WM-URD (n=112)	62.1%		0.874 (0.6-1.3)	0.480	30.3%		1.126 (0.7-1.7)	0.598
FMMT (n=52)	65.3%		0.909 (0.4-1.9)	0.795	18.5%		1.182 (0.4-3.2)	0.746
Autologous HCT (n=104)	44.7%		1.370 (0.9-2.0)	0.091	51.0%		1.962 (1.3-3.0)	0.002*
PM-URD (n=41)	36.8%		1.638 (1.0-2.6)	0.033*	33.9%		1.412 (0.8-2.6)	0.273
Acute GVHD > Grade II								
No (n=381)	59.0%	0.152			33.3%	0.043*		
Yes (n=180)	52.1%				24.6%			
Chronic GVHD (≥Moderate)								
Yes (n=140)	66.4%	<0.001*	1	-	15.4%	<0.001*	1	-
No (n=421)	53.6%		1.649 (1.2-2.3)	0.005*	35.6%		2.704 (1.6-4.5)	<0.001*

*p< 0.005

Abbreviation: HR, hazard ratio; HCT, hematopoietic cell transplantation; BHAC, N4-behenoyl-1-β-D-arabinofuranosyl cytosine; CR, complete remission; MAC, myeloablative conditioning; RIC, Reduced intensity conditioning; TBI, total body irradiation; MSD, matched sibling donor; WM-URD, well-matched unrelated donor; FMMT, familial mismatched transplantation; PM-URD, partially-matched unrelated donor; GVHD, graft-vs.-host disease; Mod, moderate;

## DISCUSSION

In the current study, we observed favorable treatment outcomes with FMMT compared to standard donor types. FMMT showed the lowest CIR rate with favorable OS and DFS rates that were comparable to MSD-HCT. The clinical outcomes of MSD, WM-URD, and FMMT were all similar in terms of OS, DFS, CIR, and NRM rates. These data suggest that frontline FMMT can be a feasible choice when MSD is unavailable prior to an URD search. In several previous studies, FMMT was compared retrospectively to chemotherapy alone, as well as WM-URD and PM-URD transplantation [19, 32–34], all of which indicated that frontline FMMT might be a good alternative choice for intermediate- to poor-risk AML. However, the limitations of a retrospective design, short follow-up duration, and different treatment modalities using post-HCT cyclophosphamide made this conclusion uncertain. Recently, a prospective multicenter clinical trial comparing the outcomes of FMMT and MSD in treating intermediate- to poor-risk adult AML in CR1 was performed. Wang et al. reported that the clinical outcomes were similar between the 2 groups and that the 3-year OS, DFS, CIR, and NRM rates for FMMT were 79%, 74%, 15%, and 13%, respectively [25]. They suggested that FMMT was a valid alternative for intermediate- to poor-risk AML patients in CR1 when MSD is not available. However, the study also had the limitations that the patients could not be randomized for ethical and practical reasons and that there were several imbalanced features, such as age and the proportion of poor-risk patients, between the two groups. Our data showed that the 5-year OS, DFS, CIR, and NRM rates for FMMT were 65.1%, 65.3%, 18.5%, and 15.7%, respectively, which were similar to the results from MSD and WM-URD. The intermediate-risk and poor-risk subgroup analysis, as well as the multivariate analysis, confirmed the positive clinical outcomes for FMMT. Based on these results, we are now conducting a prospective clinical trial evaluating the outcomes of FMMT versus WM-URD transplantation (#NCT01751997).

For FMMT, we applied a novel conditioning regimen consisting of intermediate-dose TBI (800 cGy), fludarabine, busulfan, and low-dose ATG, along with a CD34+ stem-cell dose of at least 5.0 × 10^6^ cells/kg, which, as reported previously, showed a prompt and sustained engraftment with no graft failure [19, 35, 36]. This regimen was used in 80 patients comprising all risk karyotypes and showed a low NRM (12.2%) rate and good OS when patients were in CR1 before HCT [35]. Although FMMT had a high incidence of acute GVHD ≥ grade III and CMV reactivation (which is significantly higher than in Western reports due to a high prevalence of CMV infection in Korea) similar to PM-URD, most of the incidences were safely managed. The main limitation of the analysis was the shorter median follow-up duration compared to the other donor types since FMMT was actively performed only after 2008.

In our transplantation center, PM-URD was the primary alternative donor type until 2012, but FMMT is now preferred over PM-URD. The poorer outcomes of PM-URD have been reported in several studies [22, 37], but few studies compared the outcomes of PM-URD and standard donors in adult AML. Our data support the previous results from the Center for International Blood and Marrow Transplant Research data, which showed poorer survival outcomes and significantly higher NRM rates with PM-URD [18]. One difference in our data was that the 12 patients with the poor-risk karyotype who received PM-URD transplants showed a high relapse rate (*n* = 8, 61.1%), which was not significantly different compared to that of AUTO-HCT. This result might be biased by the small number of patients in the PM-URD group who had an extremely poor karyotype including complex karyotype (*n* = 5), monosomal karyotype (*n* = 2), chromosome 3 or 5 abnormalities (*n* = 4), and t(6;11) (*n* = 1). In addition, 9 of those patients were transplanted before 2008. Nevertheless, our data suggests that FMMT can be considered prior to PM-URD, especially in cases with an urgent need for HCT. Additionally, if PM-URD is required, the dose of ATG, methotrexate, or calcineurin inhibitor should be modified to prevent severe GVHD, and CMV reactivation should be closely monitored.

Notably, our data revealed that AUTO-HCT showed acceptable OS and DFS rates, characterized by the lowest NRM rate and the highest CIR rate compared to standard allogeneic-HCT, especially in the intermediate-risk subgroup. Previous clinical trials that showed a longer duration of recovery evaluated AUTO-HCT using BM-derived progenitor cells, but recent studies have shown that PB autografts have a lower NRM rate with early hematopoietic recovery. Therefore, if we are considering AUTO-HCT, our present goal is a reduction of the relapse rate, which requires a good candidate for AUTO-HCT and a leukemia-free graft. Recent data revealed that AUTO-HCT showed a lower relapse rate compared to consolidation chemotherapy alone [38, 39], and a lower NRM rate compared to allogeneic-HCT [16]. However, our data suggests that we should avoid AUTO-HCT and consider allogeneic-HCT from standard donors or FMMT for poor-risk AML patients. Improved supportive care and techniques for stem cell mobilization may expand the application of AUTO-HCT, and post-HCT maintenance therapy can be used for the prevention of relapse [40]. We identified 52 (50%) relapsed patients among the 104 patients treated with AUTO-HCT. Among the 52 relapsed patients, 12 were treated with allogeneic-HCT and 6 (50%) of those patients are alive without relapse. However, failure to prior treatment was basically refractory to the salvage chemotherapy, and our data included only a small number of patients with possible selection bias although the result was superior to the OS rates (4-20%) of previous reports [41, 42].

We have another alternative donor source, which was not used in this study. Cord blood transplantation (CBT) has been used in patients with acute lymphoblastic leukemia and AML, and many reports showed encouraging treatment outcomes compared to URD-HCT, especially to PM-URD [43, 44]. In addition, since a recent study suggested that CBT showed similar clinical outcomes compared to FMMT [45], we may use both CBT and FMMT as an alternative donor type for patients lacking HLA-matched donors when allogeneic-HCT cannot be delayed. In our center, CBT is used for patients who relapse after FMMT, but comparative clinical trials, including qualified cord blood cell dose and optimization of pre-conditioning regimen, should be designed in near future.

Although this study is a retrospective analysis of a heterogeneous cohort over a wide transplant time period, all of which may induce bias, our observations are based on a consistent treatment strategy, including pre-HCT chemotherapy, donor searching sequence, conditioning regimens, immunosuppressive agents, and supportive care, without significant change over time. In addition, since molecular markers were not analyzed for all patients due to unavailable data, some favorable-risk CN-AML patients might be distributed in the intermediate-risk group in this study. However, the proportion would be very small and we reasonably showed the clinical outcomes of all possible donor types simultaneously. In conclusion, our data suggests that FMMT can be a good alternative for intermediate- to poor-risk adult AML patients, and we also identified the possible use of AUTO-HCT for selected patients in the intermediate-risk subgroup. These results should be validated in large prospective studies in the future.

## MATERIALS AND METHODS

### Enrolled patients

We initially found 904 adult AML patients (median age: 40 years old, range: 16-69) who underwent HCT treatment at the Catholic Blood and Marrow Transplantation Center of South Korea between 2002 and 2013. All patients were diagnosed morphologically by bone marrow (BM) aspiration and biopsy samples followed by immunophenotypic and cytogenetic analysis. For karyotyping, at least 20 metaphases from BM cells were analyzed using the GTG banding method after 24 or 48 h of unsynchronized culture, and the International System for Cytogenetic Nomenclature (ISCN) [46] and National Comprehensive Cancer Network [47] were used as guidelines for classification. As HCT is not regarded as a standard therapy for favorable-risk AML patients, we excluded 203 patients with favorable-risk molecular cytogenetics. In addition, we excluded patients who were treated with induction chemotherapy with reduced intensity due to age and severe comorbidity (*n* = 24). To include only patients in their first remission before HCT, we also excluded non-remission patients (*n* = 24), patients in their second remission (*n* = 27) before HCT, and patients treated with second HCT (*n* = 50). The final group contained 561 patients with intermediate- or poor-risk molecular cytogenetics who underwent HCT in their first remission after intensive chemotherapy. Among them, there were 417 patients in the intermediate-risk group and 144 patients in the poor-risk group (Figure 1). This research was conducted in accordance with the Institutional Review Board and Ethics Committee guidelines of the Catholic Medical Center (KC16RISI0002) and the principles of the Declaration of Helsinki.

### Molecular studies

All molecular studies were performed using initial BM samples at diagnosis. We screened 28 genetic aberrations using multiplex reverse-transcriptase polymerase chain reaction (RT-PCR) using the HemaVision Kit (DNA Technology, Aarthus, Denmark). Mutations and expression of *NPM1* were determined by real-time quantitative (RQ)-PCR using the *NPM1* MutaQuant™ kit (Ipsogen, Marseille, France), and the *FLT3-*ITD mutation was evaluated with multiplex allele-specific PCR (ABSOLUTE™ *FLT3* TKD/ITD PCR; Biosewoom, Seoul, Korea). *C-kit* mutations were detected using melting curve analysis using RT-PCR (Real-Q C-KIT screening kit and D816muta-ID kit, Biosewoom, Korea), which can detect the *c-kit* mutations located at Asp816 (D816) and Asn822 (N822K) in exon 17. These molecular studies began after 2008, and many patients from before that time did not have BM samples available for the molecular studies. Those patients were stratified only with karyotype results.

### Chemotherapy and transplantation procedure

All patients were treated according to our standard protocol, consisting of ‘3+7’ idarubicin (IDA, 12 mg/m^2^) plus ARA-C (100 mg/m^2^ continuously infused for 24 hours) or Behenoyl cytarabine (BHAC, 300 mg/m^2^), an analog of cytarabine, for remission-induction chemotherapy. After achieving CR, one or two consolidation chemotherapies were administered. Our standard consolidation chemotherapy consisted of ‘3+5’ mitoxantrone (12 mg/m^2^ iv) or IDA (12 mg/m^2^) plus an intermediate dose of ARA-C (1.0 g/m^2^ iv bid), which were alternated. In patients who were treated with BHAC for induction chemotherapy, BHAC was used for consolidation instead of ARA-C. After 2010, patients were treated with only ARA-C-based chemotherapy. During consolidation, we searched for available donors for allogeneic-HCT with a preference of MSD first, followed by WM-URD, then PM-URD. Before 2005, HLA typing was done by serology, and high-resolution sequence-based typing was used after that time. Therefore, before 2005, URD-HCT was with PM-URD. In the absence of conventional donors, FMMT or AUTO were used according to the patient's and physician's choice. If a patient was a candidate for AUTO, CD34+ stem cells were collected for 3 days when the neutrophil count recovered during the course of 2 consolidation chemotherapies. For donor mobilization, we administered G-CSF subcutaneously at a dose of 10 mcg/kg/day for 4 days.

Patients who underwent HCT from MSD, WM-URD, and PM-URD received either a MAC regimen or a RIC regimen. For the MAC regimen, briefly, we administered cyclophosphamide (120 mg/kg) combined with TBI (1320 cGy) or busulfan (12.8 mg/kg). A small proportion of patients received TBI (1320 cGy) plus busulfan (12.8 mg/kg) or TBI (1200 cGy) plus ARA-C (9g/BSA) and melphalan (100mg/BSA), which is mainly used in AUTO [48]. For the RIC regimen, we administered busulfan (6.4 mg/kg) and fludarabine (150 mg/m^2^) with 400 cGy of TBI. Anti-thymocyte globulin (ATG) at a dose of 2.5 mg/kg (1.25 mg/kg on D-3 and D-2) was administered for patients receiving stem cells from PM-URD. For FMMT, we administered fludarabine (150 mg/m^2^) and busulfan (6.4 mg/kg) with 800 cGy of TBI and ATG at a dose of 5 mg/kg (1.25 mg/kg on D-4 to D-1), all of which were described previously [19, 35]. We initially recommended BM for the stem cell source, and many of the MSD agreed, but many URD preferred PB. For FMMT and AUTO, we primarily used PB as the stem cell source, but some AUTO cases concomitantly used BM due to the lack of CD34+ stem cells.

### GVHD prophylaxis and supportive care

GVHD prophylaxis was administered using a calcineurin inhibitor plus a short course of methotrexate (5mg/m^2^ for tacrolimus and 10mg/m^2^ for cyclosporine) on D1, D3, D6, and D11. We used cyclosporine for MSD-HCT and tacrolimus for both URD-HCT and FMT. Evaluation and management of acute and chronic GVHD were based on the recommendations of the National Institutes of Health [49–51]. We used acyclovir and itraconazole for prophylaxis, and ciprofloxacin was used for prophylactic gut decontamination. After engraftment, we administered cotrimoxazole for *P. jirovecii* pneumonia prophylaxis. For surveillance of CMV reactivation, we checked RQ-PCR for CMV DNA after neutrophil engraftment and monitored for CMV reactivation twice a week until discharge. During the follow-up at the outpatient clinic, patients were monitored weekly or biweekly until the cessation of the immunosuppressive drugs. According to the CMV RQ-PCR level, risk-adapted preemptive ganciclovir therapy was conducted to prevent CMV disease. Patients were classified into low- and poor-risk groups according to both HCT type and the grade of GVHD based on our previous protocol [52, 53].

### Statistical analysis

In this study, we divided patients into 5 groups according to the HCT donor types and compared the treatment outcomes. Between the groups, we compared OS, DFS, CIR, and NRM rates in association with the incidence of GVHD and CMV reactivation. All categorical variables were compared using Chi-squared analysis and continuous variables were assessed with the Student's *t*-test and one-way analysis of variance. OS and DFS was calculated using Kaplan-Meier analysis, and log-rank analysis was used to evaluate differences between the groups. OS represented the proportion of people who were alive at a specified time from the date of allogeneic-HCT and DFS took into account death, relapse, lost to follow-up as the result of treatment complications. CIR and NRM were calculated by cumulative incidence estimation treating non-relapse deaths and relapse as competing risks, respectively, and compared using the Gray test [54]. Survival hazard ratio was calculated using Cox's proportional model. All statistical analyses were performed using SAS 9.2 software (SAS Institute, Inc., Cary, NC) and R software (version 2.15.1, R foundation for statistical Computing, 2012). Statistical significance was determined with *p*-value < 0.05.
